# Photo-Sintered Silver Thin Films by a High-Power UV-LED Module for Flexible Electronic Applications

**DOI:** 10.3390/nano11112840

**Published:** 2021-10-25

**Authors:** Minha Kim, Hongsub Jee, Jaehyeong Lee

**Affiliations:** Department of Electrical and Computer Engineering, Sungkyunkwan University, Suwon 16419, Korea; pratice1dea@naver.com (M.K.); hsjee@skku.edu (H.J.)

**Keywords:** silver thin film, photo-sintering, ultraviolet light emitting diode (UV-LED), low temperature process, flexible substrate

## Abstract

In recent printed electronics technology, a photo-sintering technique using intense pulsed light (IPL) source has attracted attention, instead of conventional a thermal sintering process with long time and high temperature. The key principle of the photo-sintering process is the selective heating of a thin film with large light absorption coefficients, while a transparent substrate does not heat by the IPL source. Most research on photo-sintering has used a xenon flash lamp as a light source. However, the xenon flash lamp requires instantaneous high power and is unsuitable for large area applications. In this work, we developed a new photo-sintering system using a high-power ultraviolet light emitting diode (UV-LED) module. A LED light source has many merits such as low power consumption and potential large-scale application. The silver nanoparticles ink was inkjet-printed on a polyethylene terephthalate (PET) and photo-sintered by the UV-LED module with the wavelength of 365 and 385 nm. The electrical resistivity as low as 5.44 × 10^−6^ Ω·cm (just about three times compared to value of bulk silver) was achieved at optimized photo-sintering conditions (wavelength of 365 nm and light intensity of 300 mW/cm^2^).

## 1. Introduction

For decades, printed electronics technology has been developed with materials like metal nanoparticles ink, non-contact technology like drop-on-demand inkjet technology for printing materials on any substrate, and post-treatment process in order to obtain good properties from materials [1,2,3,4,5,6,7,8]. In particular, a new frontier of printed electronics is to print flexible and stretchable electronic devices for wearable electronics [9,10,11]. These developments are expected to offer many advantages such as flexibility, low cost, and simple process compared with to existing processes in industry. Recently, thanks to the developments, printed electronics have applied to radio-frequency identification (RFID) tags, display, and various types of sensors [12,13].

Conductive inks based on highly concentrated metal nanoparticles have special physical properties different from the bulk metal. For example, the metal nanoparticles with high surface to mass ratio have high absorption and low melting point compared to the bulk metal. Generally, since the metal nanoparticles inks could be polarized by electromagnetic wave propagating along metal-solvent dielectric boundary, these particles have plasmon resonance peaks depending on particle size and shape, dielectric constant of external solvent and these particles, and substrate optical properties [14]. In particular, the metallic particles can be shown to have stronger absorption than the light incident at ultraviolet frequencies [15].

In general, traditional thermal processes are very difficult to apply to flexible polymer substrates since high temperature would destroy the substrate and inert gas for good electrical properties. Therefore, various techniques have been used to sinter silver nanoparticles, including chemical self-sintering [16,17], electrical [18,19,20], infrared [21,22,23,24], laser [25,26,27], microwave [28,29,30], plasma [31,32,33,34], and photo sintering methods [35,36,37,38,39,40,41]. Among these post-annealing processes, photo-sintering has been the center of attention as a new method process without significantly heating the substrate [42]. Recently, photo-sintering technology has been applied to a variety of nanoparticles [43,44] and nanowires [45,46,47] for low-temperature processing. The key principle is the selective heating of a thin film composed of metal nanoparticles with strong absorption property, which is an essential parameter needed to increase the energy transfer from light, while the temperature of transparent substrate does not rise up by an intense pulsed light (IPL) source. Photo-sintering has very strong merit in that the process is very easy and is carried out at room temperature under air atmosphere conditions [48]. Many researchers have used a xenon flash lamp with wide-range wavelengths for photo-sintering and investigated the effects of flashing frequency and light intensity on the properties of materials. However, IPL needs an instantaneous high power, which is one of the main reasons for very expensive system cost, and has the problem of uniform light intensity for large-area applications.

Therefore, in this work, we invented a new photo-sintering system using high-power ultraviolet light emitting diode (UV-LED) modules with different wavelengths for sintering silver nanoparticle thin film ink-jet printed on the PET substrate. When using high-intensive LED, easily-controllable sintering time, needless high-voltage, more-efficient power consumption, and better durability, could be much friendlier to large-scale industry. Effects of photo-sintering parameters such as light intensity, irradiation duration, and wavelength of UV-LEDs on the electrical properties of Ag thin films were investigated.

## 2. Experimental Details

**Figure 1:**
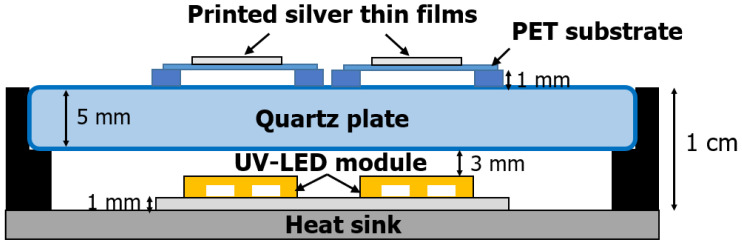
Schematic diagram of the high-power UV-LED module system.

**Figure 2:**
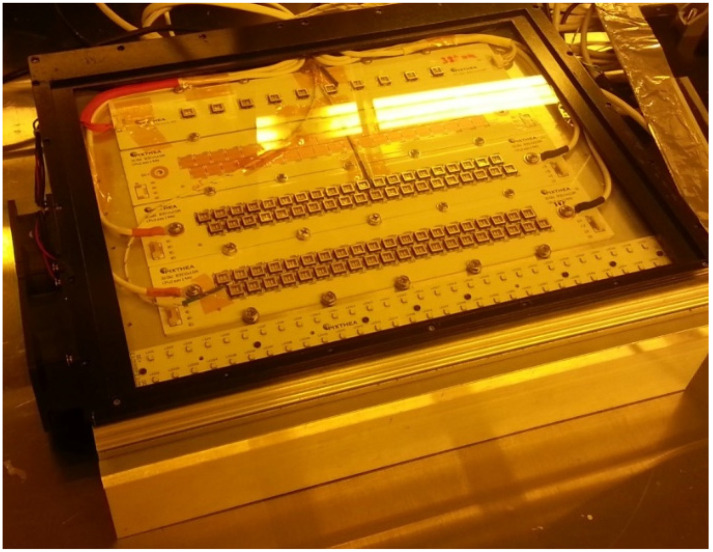
Prototype of the UV-LED module with a heat sink.

Silver nanoparticles ink was obtained commercially: an ethylene glycol-water based silver nanoparticles ink (JS–B25P, Novacentrix, Austin, TX, USA), with a particle size of 102.3 nm (93.5%), 14.53 nm (6.5%), and average particle size 71.9 nm. The initial content of silver in the ink was 25%. The silver ink viscosity of 4.60 cP at 22.0 °C and the surface tension of 31.2 dynes/cm were suitable for Epson Workforce 30 (Epson, Suwa, Japan), a high resolution and performance piezoelectric printer. The ink was printed on Novel^TM^ (Novacentrix, Austin, TX, USA) PET substrate with the area of 1 × 1 cm^2^. The photo-sintering of Ag thin films was performed by the UV-LED module. The module composing of 2 × 20 arrays was made of high-power UV-LEDs with two wavelengths of 365 nm (NC4U133A(T), Nichia, Anan, Japan) and 385 nm (NC4U134A(T), Nichia, Japan), as shown in Figure 1 and Figure 2. The electric circuits consisting of 2-series by 20-parallels connection was suitable for DC power supply, PAX 35–20 (Kikusui, Yokohama, Japan) with voltage range 0 to 35 V and current range 0 to 20 A. The light intensity of the module was controlled by adjusting the current of the DC power supply. The light intensity per unit area (W/cm^2^) corresponding to the current was confirmed by a UV radiation measurement (UIT–150, Ushio, Tokyo Japan) (Table 1). All photo-sintering processes were performed at 20 °C, in air atmosphere, and under 40% humidity conditions. Samples were placed in a position 1 mm away from the quartz plate, which was intended to protect the UV-LED module. In addition, the silver thin film was positioned upward since a solvent evaporated from the silver ink during sintering clings to the quartz plate. For comparison, a thermal sintering of silver film was performed in the vacuum dry oven.

The thermogravimetric curves of the silver ink were analyzed by a thermogravimetry differential thermal analyzer (TG–DTA, TG/DTA7300, SII Nanotechnology Inc., Tokyo, Japan) under nitrogen gas atmosphere. Optical spectra of the silver–ink was obtained with a UV-visible absorption spectrometer (UV-VIS, S–3100, Scinco, Seoul, Korea). The sheet resistance was measured by a four-point probing system (CRESBOX, Napson Corporation, Tokyo, Japan). The crystal structure analysis of the silver film deposited on the PET substrates was investigated by X-ray Diffractometer (XRD, DB Advance, Bruker, Billerica, MA, USA). The microstructures and the surface morphologies of the silver thin films were examined by field emission scanning electron microscopy (FE–SEM, JSM6700F, JEOL, Tokyo, Japan). The surface temperature of the silver film during photo-sintering was measured by four-input thermometer (TES–1384, TES, Taipei, Taiwan).

## 3. Results and Discussion

**Figure 3:**
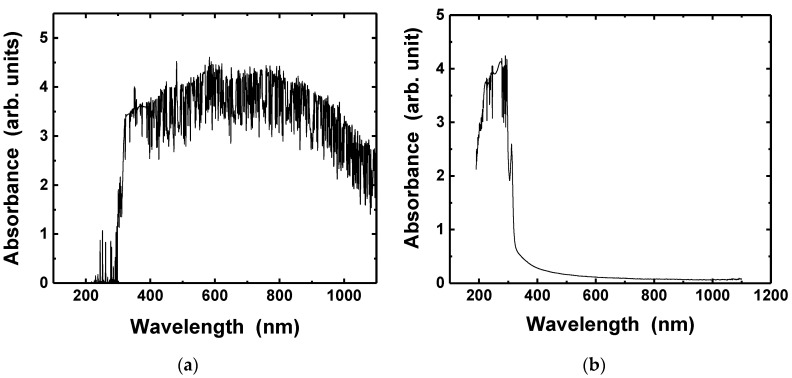
Absorbance spectra of the silver nanoparticle ink (**a**), PET substrate (**b**).

**Figure 4:**
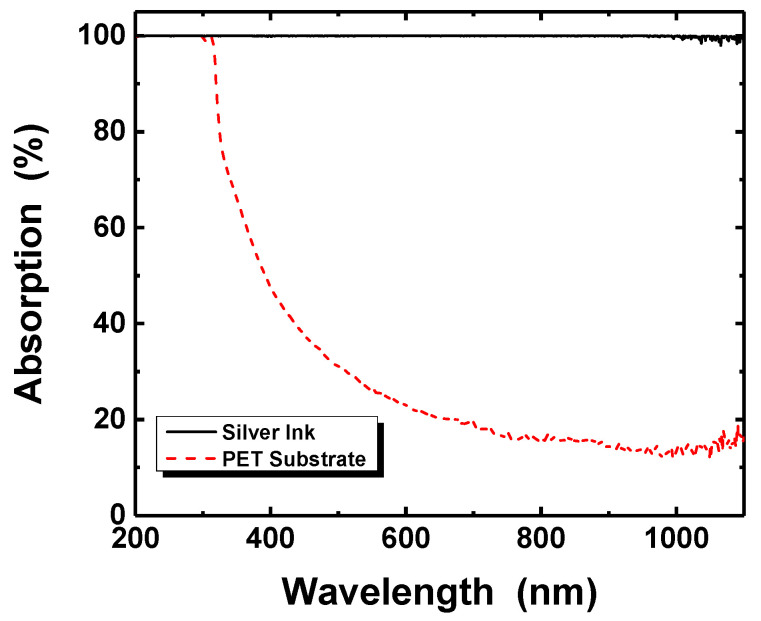
Optical absorption spectra of Ag nanoparticle ink and PET substrate.

Figure 3 shows the optical absorbance spectra of the silver nanoparticle ink and the PET substrate. The UV-visible spectrophotometer measures the optical absorbance. The absorption can be calculated from the following equation.

(1) $$A(\%)=(1-10^{-\alpha })\times 100$$

where *A* is the absorption and *α* is the absorbance [49,50]. The silver ink exhibited very high absorption (near 100%) at the wavelength of 365 and 385 nm, while the PET substrate appeared with optical absorption over 50%, as shown in Figure 4. The light absorption of the silver nanoparticles may be attributed to the localized surface plasmon resonance (LSPR), which is the resonant photon-induced coherent oscillation of charge at the metal–dielectric interface when the photon frequency matches the natural frequency of the metal surface electrons oscillating against the restoring force of their positive nuclei [51].

**Figure 5:**
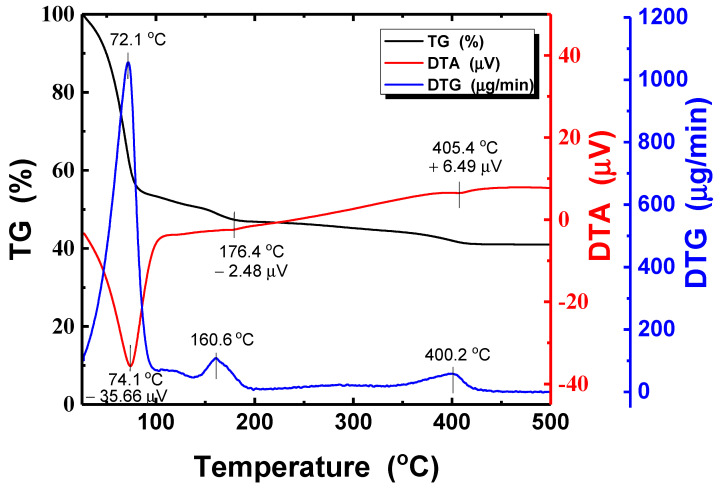
Thermogravimetric (TG), differential thermal analysis (DTA), and derivative thermogravimetric (DTG) of the silver nanoparticle ink.

Figure 5 displays the TG, DTA, and DTG curves of the silver ink in the temperature range from 25 to 500 °C. The heating rate was 10 °C/min. DTA data indicate endothermic reactions at two specific temperatures (74.1 and 176.4 °C) and exothermic reaction at one specific temperature (405.4 °C), respectively. First endothermic reaction at 74.1 °C with an initial weight loss of 45% may be the result of evaporation of a residual solvent, water. Second endothermic reaction at 176.4 °C is closely related to a residual solvent, ethylene glycol with the boiling point of 197.3 °C. There is endothermic reaction until approximately 240 °C, according to DTA results. Over this temperature, most of the solvent in the silver ink can be expected to disappear by heating. However, interestingly there is still a little weight loss from 240 to 405.4 °C (exothermic reaction temperature). It is postulated that residues from some surfactants in the ink are decomposed thermally. From DTG results, three main ingredients in the ink were confirmed. The two peaks at 72.1 and 160.6 °C correspond to water and ethylene glycol, respectively, and the weak peak at 400.2 °C might be associated with a little surfactant. In particular, at temperature near the boiling point of ethylene glycol, its dehydration, following Equation (2), could happen to reduce metal oxide nanoparticles [52].

(2) $$\begin{array}{l} {2\mathrm{CH}}_{2}\mathrm{OH}-{\mathrm{CH}}_{2}\mathrm{OH}\overset{{-H}_{2}O}{\to }2{\mathrm{CH}}_{2}\mathrm{CHO}\\ \overset{M(\mathrm{II})}{\to }{\mathrm{CH}}_{3}-C-C-{\mathrm{CH}}_{3}+H_{2}O+M\\ \;\;\;\;\;\;\;\;||\;\;\;\;||\\ \;\;\;\;\;\;\;\;\;\;O\;\;\;\;O \end{array}$$

For good electrical properties, all solvents in the silver thin film must be eliminated. Therefore, at least, over ethylene glycol’s dehydration temperature (160 °C), post-annealing treatment should be carried out for crystallization. 

**Figure 6:**
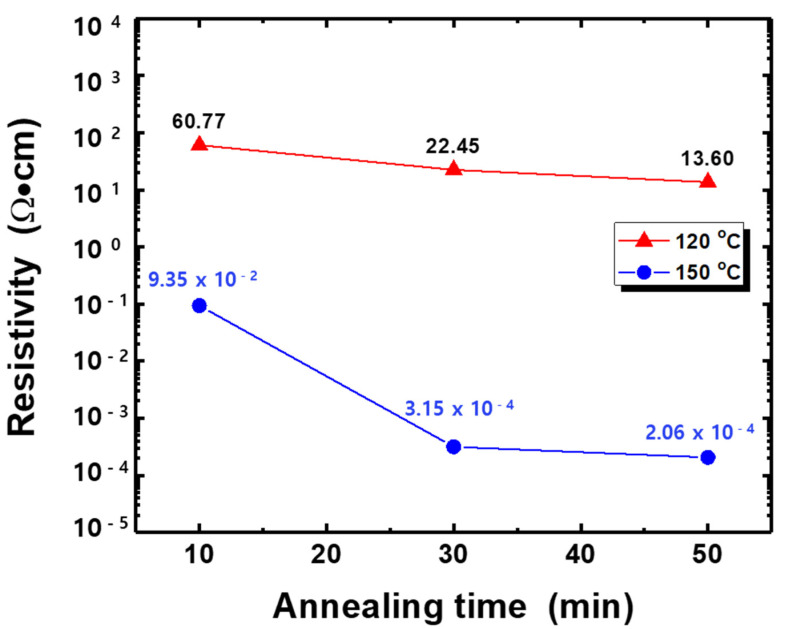
Electrical resistivity of silver thin films thermally treated at different temperatures and for various durations. The film thickness was about 700–800 nm.

From TG/DTA analysis, we knew at least the temperature of 160.6 °C might be required for post-treatment of the silver ink. Figure 6 shows the sheet resistance of silver thin films annealed at different temperatures and for various durations in the vacuum. When the process temperature was over 180 °C, PET substrate became bent and damaged. On the other hand, at the annealing temperature lower than 90 °C, the films exhibited very high sheet resistance (over 2 MΩ/sq.), although process time was several hours. 

The change of the resistivity in the temperature range from 120 to 150 °C may be attributed to dehydration of ethylene glycol. Under 90 °C, only water in the silver film is evaporated simply without any chemical reaction. There was still ethylene glycol in the silver film, and also the temperature might have too little energy to crystallize silver nanoparticles. When the temperature increased to 120 °C, some chemical reactions like ethylene glycol’s dehydration and evaporation could occur in the silver film. However, it does not seem enough to crystalize silver film as can be expected from high resistivity. As the temperature further increased to 150 °C, the resistivity decreased significantly. Although the temperature was not over 160 °C (ethylene glycol’s dehydration temperature), the film could evaporate ethylene glycol for a long time, and there might be chemical reaction, which looks capable of crystalizing it. Ag thin film thermally treated at 150 °C for 50 min exhibited the resistivity of 2.06 × 10^−4^ Ω·cm.

**Figure 7:**
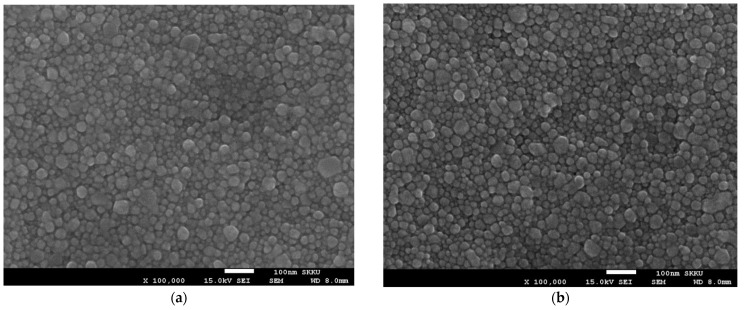

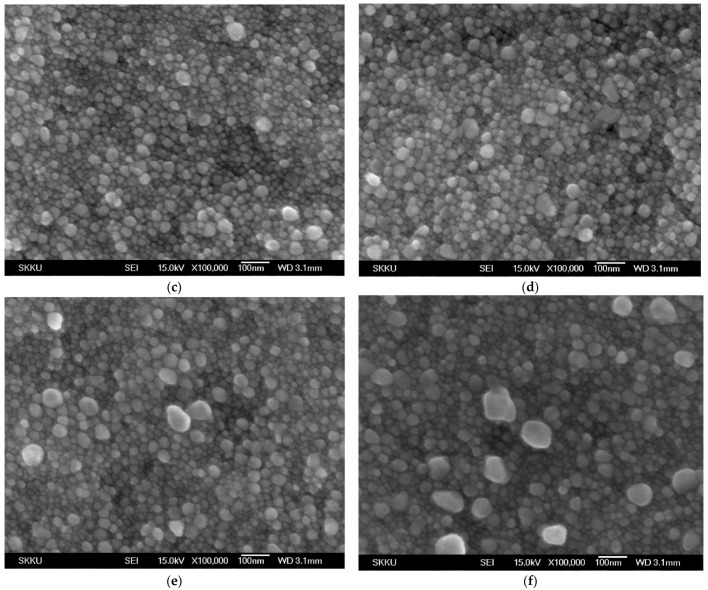
SEM images showing the surface morphology of thermally treated Ag films: as-deposited (**a**), annealed at 90 °C (**b**), 120 °C (**c**), 150 °C (**d**) for 10 min. The sample (**e**,**f**) were annealed at 150 °C for 30 and 50 min, respectively.

Figure 7 displays the surface morphologies of silver films thermally treated at different temperatures and various durations. There is little change of the surface structure when the film was thermally treated for a short duration (i.e., 10 min), regardless of the temperature. However, some large size nanoparticles appeared on the film surface as the treatment duration became longer, especially 50 min.

**Figure 8:**
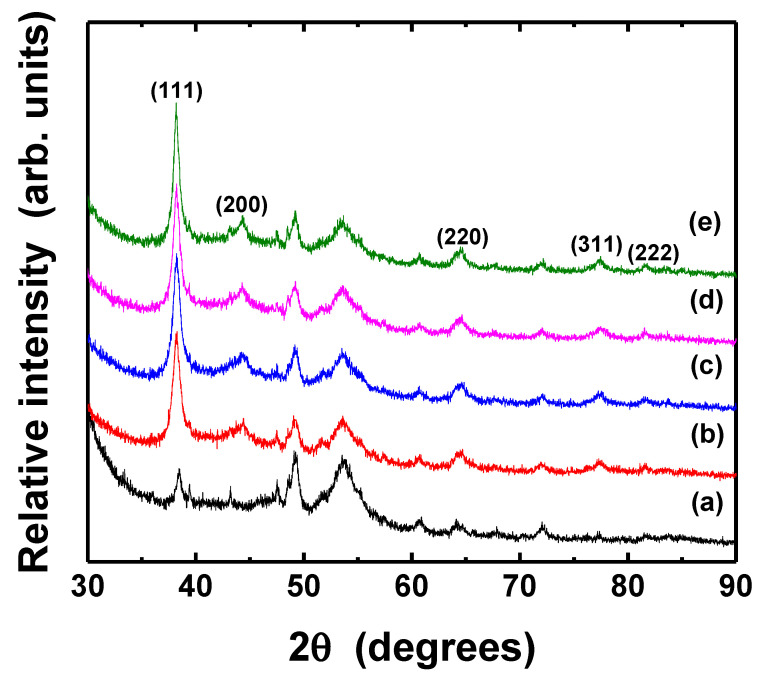
XRD patterns of Ag thin films thermally treated at different temperatures: (**a**) PET substrate, (**b**) as-deposited, (**c**) 90 °C, (**d**) 120 °C, (**e**) 150 °C, respectively. The pot-treatment duration was 10 min.

**Figure 9:**
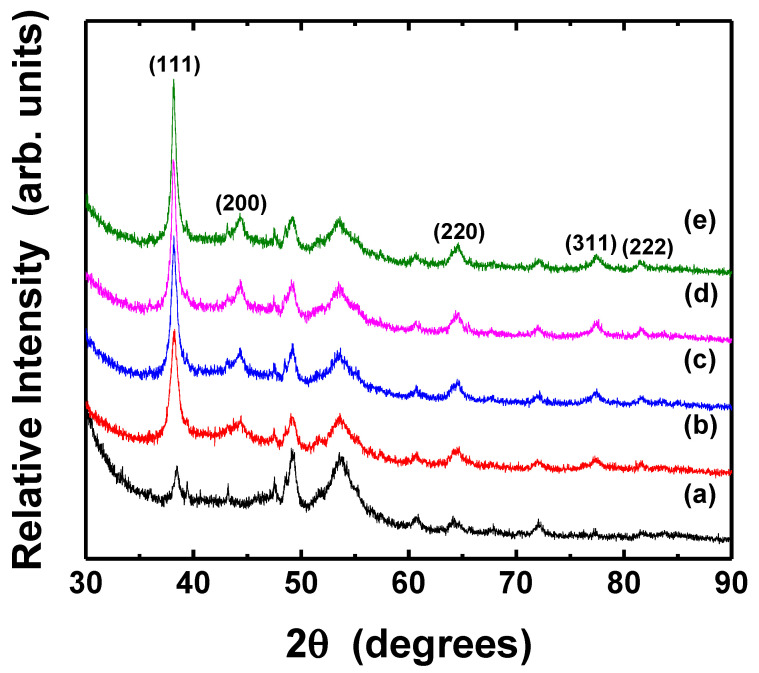
XRD patterns of Ag thin films post-treated for various durations: (**a**) PET substrate, (**b**) as-deposited, (**c**) 10 min, (**d**) 30 min, (**e**) 50 min, respectively. All samples were thermally treated at 150 °C.

The crystal structure and the grain size of thermally treated Ag thin films were analyzed by XRD, as shown in Figure 8 and Figure 9. The mean particle diameter in the silver films was calculated from the diffraction peak of Ag (111) plane as following Scherrer Equation (3) [53].

(3) $$D=\frac{K\lambda }{\beta \cos\theta }$$

where *λ* is the X-ray wavelength (Cu Kα, 0.154 nm) in nanometer (nm), *β* is the full width at half-maximum (FWHM) in radians, *θ* is the diffraction angle, and *K* is a constant related to crystallite shape, generally taken as 0.9. All samples exhibit the several diffraction peaks corresponded to (111), (200), (220), (311), and (222) planes of pure crystalline face-centered cubic (fcc) silver, respectively [54]. The peak of (111) plane was dominant, although Ag (111) and (220) peaks overlapped with XRD peaks associated with PET substrate. The intensity of the peak corresponding to the silver (111) was significantly higher than that of the PET substrate; however, the (220) peaks of silver in the as-deposited and thermally treated silver films also had apparently identical intensities. The peak intensity of (111) plane of Ag films increased slightly after the post-treatment at the different temperatures and various durations, suggesting improvement of crystallinity, although the morphology of the particles only had little changes. Table 2 shows the grain size of silver films thermally treated at different conditions. The grains in the films became bigger as the temperature and duration increased, as can also be seen in Figure 7.

**Figure 10:**
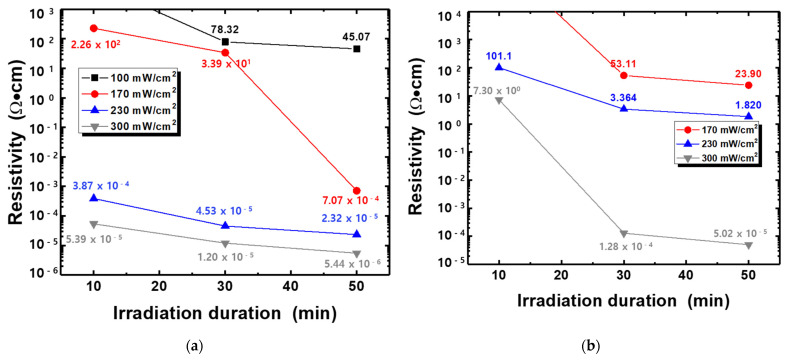
Dependence of the light intensity and irradiation duration of UV-LED module on the electrical resistivity of Ag films: (**a**) 365 nm, (**b**) 385 nm.

Figure 10 shows the dependence of the light intensity and irradiation duration of UV-LED modules on the resistivity of Ag films. For a wavelength of 365 nm, the electrical characteristic of Ag films was improved when the silver film was photo-sintered at higher light intensity and for longer irradiation duration. In particular, significant reduction of the resistivity was observed on the samples irradiated with a light intensity of 300 mW/cm^2^. The minimum resistivity of 5.44 × 10^−6^ Ω·cm (just about three times compared to value of bulk silver) was obtained from the optimum photo-sintering conditions of 300 mW/cm^2^ and 50 min. Similar results were obtained for a wavelength of 385 nm, even though the resistivity exhibited larger values in all samples, suggesting the shorter wavelength of UV-LED are more effective for better electrical performance. The minimum resistivity of 5.44 × 10^−6^ Ω·cm (sheet resistance of 72.6 mΩ/sq), comparable value from Ag films photo-sintered with IPL [55], was obtained from the optimum photo-sintering conditions (300 mW/cm^2^ and 50 min). Comparing to values of thermally sintered silver film (Figure 5), the films irradiated with the UV-LED modules appeared to have better electrical conduction, regardless of the wavelength. The significant changes in the electrical resistivity of Ag thin films depending on the sintering conditions are attributed to several reasons. Dziedzic et al. [56] suggested that when the curing temperature got higher, the mass and thickness of the conductive pillars in the carbon/polyesterimide (PEI) composite films decreased, resulting in lower electrical resistance. However, there was no large difference in the thickness of Ag films depending on the curing temperature or the sintering methods (thermal and photo-sintering), although the as-prepared film thickness could not be measured. In addition, no significant changes appeared in the crystal structure (XRD analysis in Figure 8 and Figure 9). Therefore, the improvement of the electrical conductivity after thermal- and photo-sintering process is due to greater grain growth as shown in Figure 7.

**Figure 11:**
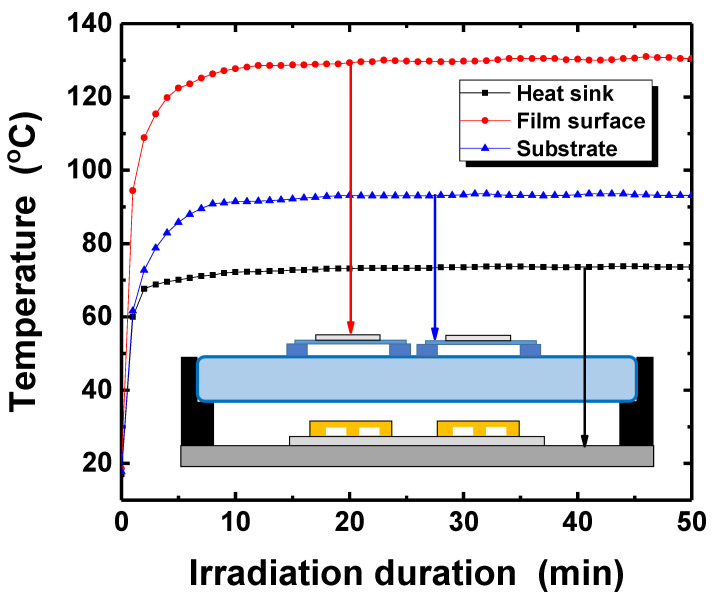
Temperature profiles of the silver film surface, the substrate, and the heat sink as a function of the irradiation duration. The light intensity of UV-LED module with the wavelength of 365 nm was 300 mW/cm^2^.

The temperature changes at different sites of the UV-LED system during irradiation were monitored (Figure 11) in order to investigate the mechanisms governing the process. When the high intensity light was irradiated on the silver nanoparticles film, the film surface and the substrate temperature were saturated to approximately 90 and 130 °C within 10 min, as shown in Figure 11. The electrical characteristics of photo-sintered film is better than sample thermally annealed at 150 °C, even though the surface temperature during UV-LED irradiation is lower than 150 °C. This might be because of the excited electron resulting from UV-light irradiation. It is possible that the energetic electrons of the silver nanoparticles from the light irradiation remain in the excited state for several picoseconds as Au nanoparticles [57]. Hence, the excited electrons in the nanoparticles might activate reactant molecules, ethylene glycol, adsorbed on the Ag nanoparticles, and lead to more chemical reactions under lower temperature than its dehydration temperature [51].

**Figure 12:**
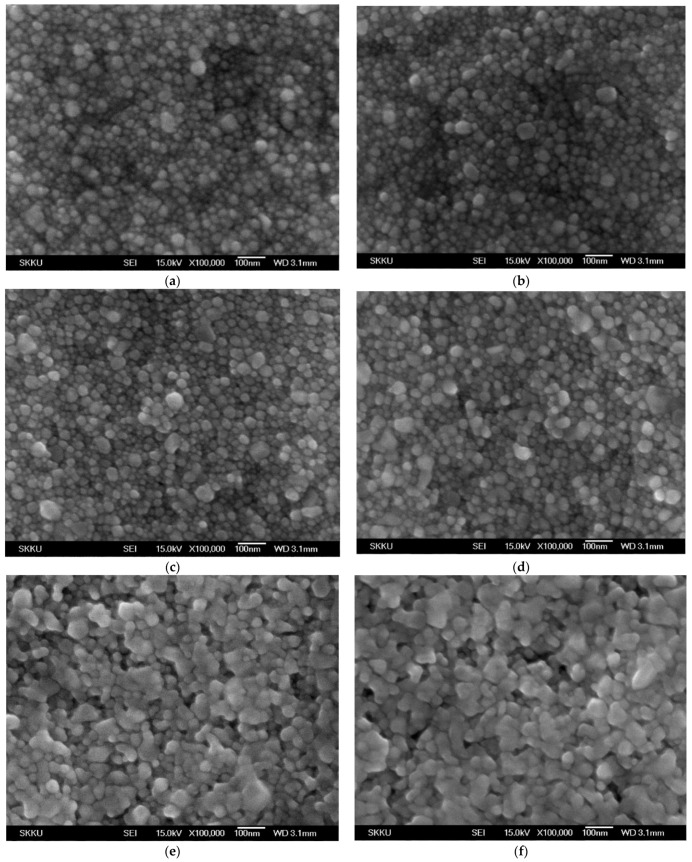
Surface morphologies of photo-sintered Ag films under various conditions: 100 mW/cm^2^ (**a**), 170 mW/cm^2^ (**b**), 230 mW/cm^2^ (**c**), 300 mW/cm^2^ (**d**) for 10 min, respectively. The sample (**e**,**f**) were photo-sintered at 300 mW/cm^2^ for 30 min and 50 min. The wavelength of UV-LED module was 365 nm.

The surface morphologies of UV-LED sintered silver films were different from those of thermally treated samples, as shown in Figure 12. There is little change on the surface when the films were irradiated for a short time (i.e., 10 min), regardless of the light intensity. As the irradiation duration further increased, coarsening of intergranular silver nanoparticles occurred and the grains got larger.

**Figure 13:**
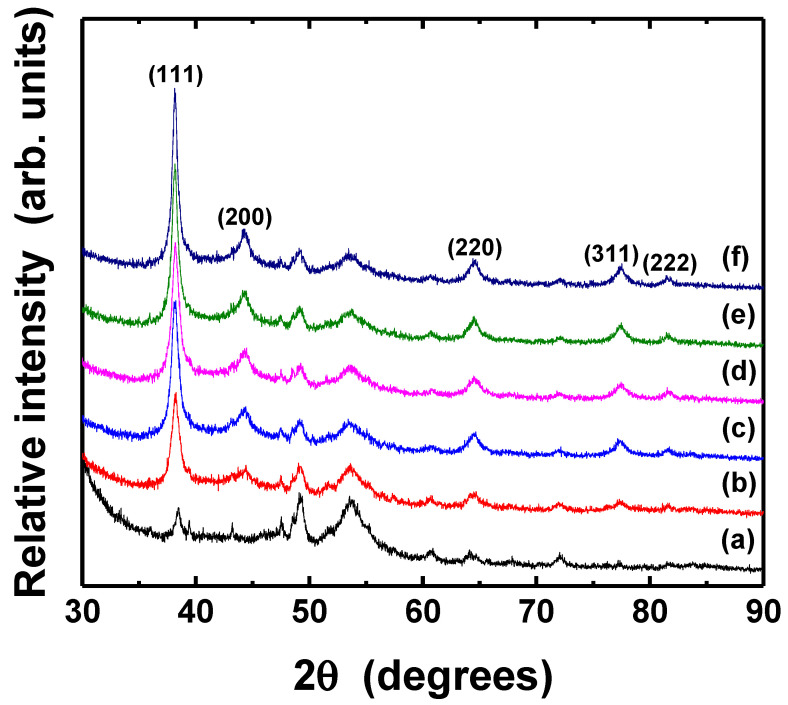
XRD patterns of Ag thin films photo-sintered at different light intensities: PET substrate (**a**), as-deposited film (**b**), 100 mW/cm^2^ (**c**), 170 mW/cm^2^ (**d**), 230 mW/cm^2^ (**e**), and 300 mW/cm^2^ (**f**), respectively. The wavelength of UV-LED module was 365 nm, and all samples were photo-sintered for 10 min.

**Figure 14:**
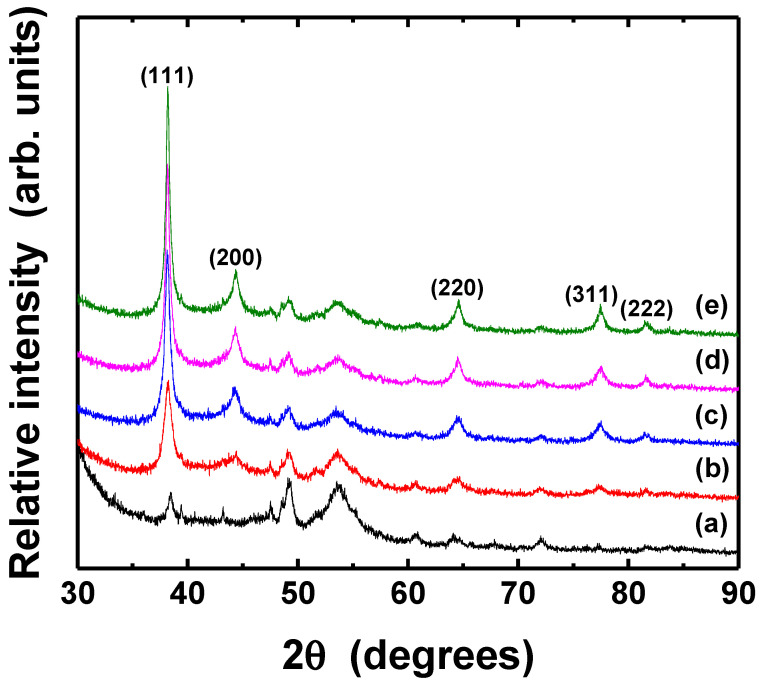
XRD patterns of Ag films irradiated for various durations: PET substrate (**a**), as-deposited (**b**), 10 min (**c**), 30 min (**d**), and 50 min (**e**), respectively. The wavelength and the light intensity of UV-LED module were 365 nm and 300 mW/cm^2^, respectively.

Figure 13 and Figure 14 show the XRD patterns of Ag thin films photo-sintered under different conditions. The (1 1 1) peak of fcc silver phase became sharper after UV-LED irradiation, indicating that the crystallinity of the film improved, regardless of photo-sintering conditions. In particular, the increase of peak intensity was prominent when the film was irradiated for 50 min (Figure 14e). The mean grain diameter of photo-sintered silver films was calculated from the Ag (111) plane peak using Scherrer equation (Table 3). The grains in the Ag films enlarged as the light intensity and irradiation duration increased. Compared to thermally sintering film, the grain size of the film photo-sintered at high power and longer irradiation (i.e., 300 mW/cm^2^, 30 and 50 min) was larger.

## 4. Conclusions

This work studied the UV-LED light sintering effects on the electrical and structural properties of silver thin films inkjet-printed on the PET substrate. For comparison, the films were also thermally sintered at different temperatures and for various durations. The TG/DTA analysis revealed that minimum temperature of 160.6 °C was required for post-annealing of Ag films. The post-treatment temperature showing good electrical performance was 150 °C and the sheet resistance reduced significantly with the thermal sintering time. Using optimized irradiation conditions like a light intensity of 300 mW/cm^2^ and an irradiation duration of 50 min, the minimum resistivity of 5.44 × 10^−6^ Ω·cm (much smaller value compared to thermally sintered Ag film) could be obtained even though the film was post-treated in air atmosphere, instead of vacuum. In addition, the UV-LED with a shorter wavelength (365 nm) was more adequate for better electrical performance. The new photo-sintering system with the high-power UV-LED module can be applicable to large area applications for various metals and semiconductor to achieve good electrical properties.

## Figures and Tables

**Table 1:** Light intensities per unit area and surface temperature of UV-LEDs at some input currents.

Wavelength (nm)	Input Current (A)	Input Current per LED (mA)	Saturation Temperature (°C)	Measured Light Intensity (mW/cm^2^)
365	1.1	55	40	106.0
365	1.7	85	48	171.1
365	2.3	115	57	230.0
365	3.1	155	69	301.0
385	1	50	33	168.1
385	1.3	65	35	226.8
385	1.7	85	37	301.8

**Table 2:** Grain size of thermally treated Ag thin films under different conditions.

Post-Treatment Conditions	(111) Plan			
	2θ (Degree)	*d* (Å)	FWHM (Degree)	Grain Size (nm)
90 °C, 10 min	38.232	2.3522	0.779	10.79
120 °C, 10 min	38.216	2.3531	0.738	11.39
150 °C, 10 min	38.194	2.3544	0.624	13.47
150 °C, 30 min	38.17	2.3559	0.535	15.71
150 °C, 5 0min	38.175	2.3556	0.497	16.91

**Table 3:** Grain size of Ag thin films photo-sintered at various light intensities and for different irradiation durations. The wavelength of UV-LED module was 365 nm.

Post-Treatment Conditions	(111) Plan			
	2θ (Degree)	*d* (Å)	FWHM (Degree)	Grain Size (nm)
100 mW/cm^2^, 10 min	38.167	2.3560	0.742	11.33
170 mW/cm^2^, 10 min	38.211	2.3535	0.736	11.42
230 mW/cm^2^, 10 min	38.157	2.3567	0.606	13.87
300 mW/cm^2^, 10 min	38.167	2.3560	0.542	15.51
300 mW/cm^2^, 30 min	38.181	2.3552	0.456	18.44
300 mW/cm^2^, 50 min	38.198	2.3542	0.393	21.39

## Data Availability

The data that support the findings of this study are available upon reasonable request.
